# Effect of Loading Rate on Tensile and Failure Behavior of Concrete

**DOI:** 10.3390/s20215994

**Published:** 2020-10-22

**Authors:** Xiaocui Chen, Liguo Sun, Wenhu Zhao, Yuan Zheng

**Affiliations:** 1 College of Energy and Electrical Engineering, Hohai University, Nanjing 211100, China; chenxc@hhu.edu.cn (X.C.); zhengyuan@hhu.edu.cn (Y.Z.); 2 College of Mechanics and Materials, Hohai University, Nanjing 211100, China; whzhao@hhu.edu.cn

**Keywords:** concrete, strain rate, dynamic strength, dynamic increase factor, three point bending test

## Abstract

Three-point bending experiments of concrete beams were conducted under the strain rate range of 10^−6^ s^−1^ and 1.5 × 10^−3^ s^−1^. A novel 3D laser scanner, Handy SCAN, was employed to detect the areas of interface, mortar and aggregate on the crack surface after the experiment. In this paper, the inhomogeneity of materials and the inertial effect were considered as the main factors in the strength enhancement of concrete together with a proposed dynamic model. With the obtained experimental results, the initial elastic modulus and tensile strength of concrete showed obvious rate sensitivity. Moreover, an empirical relationship of dynamic increase factor and strain rate was established for the strain rate range of 10^−6^ s^−1^ and 1.5 × 10^−3^ s^−1^. The contributions of aggregate and inertia effect to the dynamic enhancement of concrete strength were quantified with respect to the loading rate. The rate effect of concrete obtained by the experiments was verified by the finite element analysis on the mesoscopic scale with the model built by the three-dimensional random aggregate software.

## 1. Introduction

In civil engineering, concrete structures inevitably suffer from different type of dynamic loading during their designed lifetime, such as earthquakes. Moreover, tall buildings and bridges may suffer from wind loading. Concrete dams encounter hydrodynamic loading. Offshore platforms endure the impact of ocean waves [1,2,3]. Dynamic loading can cause the damage and collapse of these structures, which will lead to economic loss and major casualties. Hence, in the design of these structures, it is important to concern these loads, because they are unpredictable and destructive [4,5]. In addition, the dynamic performance of concrete is necessary to study. As a primary building material, concrete is rate-dependent, with its strength, stiffness and ductility subjected to the loading rate. In 1917, Abrams first observed that the concrete strength was sensitive to the loading rates in the compressive experiments with the strain rates of 8 × 10^−6^ s^−1^ and 2 × 10^−4^ s^−1^ [6]. The concept of the dynamic increase factor (DIF) was introduced to characterize the dynamic strength enhancement behavior and has been widely accepted, defined as the ratio of the dynamic strength to the quasi-static strength in the uniaxial compression or in uniaxial tension.

With the awareness of the dynamic behavior of concrete and its tremendous effect, numerous experiments have been conducted under different strain rates to investigate the relationship between the dynamic increase factor and strain rate [7,8,9]. The split Hopkinson pressure bar (SHPB) technique is often employed to identify the rate-sensitive behavior of concrete-like materials [10,11,12,13] at a range of strain rates between 10^2^ s^−1^ and 10^4^ s^−1^, as well as explosive devices for higher strain rates and other techniques [14]. Based on the extensive experimental data, several empirical equations of DIF with respect to strain rate have been proposed for concrete [15,16,17]. Brace and Jones [18] first noticed the transition from the uniaxial stress state to uniaxial strain state, because the specimen was unable to move laterally during the experiment. Qi and Qian [19] proposed a unified model of competition between the thermal activation mechanism and macro-viscosity mechanism for the brittle materials to explain the dynamic strength enhancement effect and insisted that the thermal activation mechanism was dominant at low strain rates, while the inertial force had a great influence in the high strain rate range. The results of some experiments [20,21,22] indicated that the enhancement effect was caused by the inherent material properties and lateral inertial confinement effects. Weerheijm [23] believed that the inertial effect was the main factor responsible for the rate-dependent behavior of concrete. Many factors, such as the water/cement ratio, type of aggregate, mix proportions and curing conditions, may influence the enhancement effect [24].

In this paper, the inherent material properties and the inertial effect were considered as the main contributions to the strength enhancement of concrete. Three-point bending experiments of concrete beams were conducted under different strain rates, and a novel 3D laser scanner, Handy SCAN, was employed to measure the areas of bonding interface, mortar and aggregate on the crack surface. Based on the experimental data, a relationship between the DIF and strain rate was proposed at the strain rate range of 10^−6^ s^−1^ and 1.5 × 10^−3^ s^−1^. Moreover, the contributions of aggregate and inertia to the increase in dynamic strength of concrete were studied. Later, the 3D finite element model on the mesoscopic scale was built, and the numerical simulations of the concrete beams under different strain rates were conducted based on the obtained experimental results.

The remainder of the paper is organized as follows: first, the concrete beams for the three-point bending tests and the experimental setup are introduced in Section 2. Static tests with a loading velocity of 0.1 mm/min and dynamic tests with three different loading velocities are detailed in Section 3. The experimental results emphasize the load-time curves and stress-strain curves under different loading conditions, together with the areas of different components on the crack surfaces generated by Handy SCAN. In Section 4, a relationship between the DIF and strain rate is proposed to predict the increase in strength with the increasing strain rate. Moreover, the area percentage of the aggregate is employed in this section to discuss the effect of the aggregate on the dynamic strength enhancement of concrete, and the dynamic inertia contribution to the dynamic strength was studied as well. Finally, a 3D finite element analysis of concrete beam with the loading velocities of 0.1 mm/min and 150 mm/min was conducted with the model built by the 3D-RAS (three-dimensional random aggregate software) with scanned results and the concrete mixture. Final concluding remarks are given in Section 5.

## 2. Experimental Setup

### 2.1. Test Specimens

**Figure 1:**
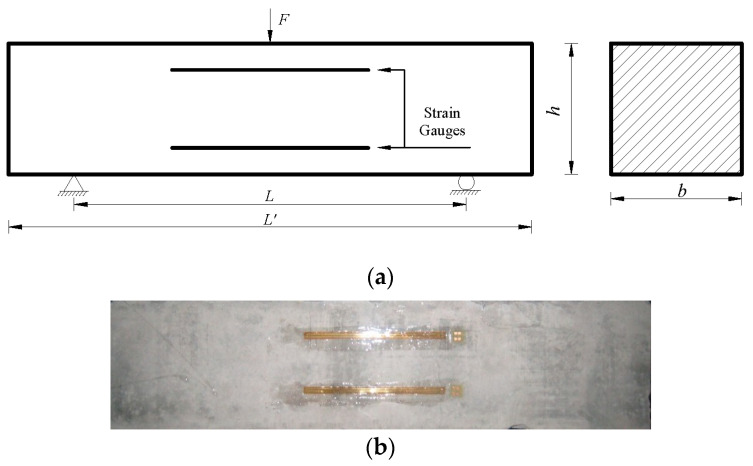
The three-point bending test (*L* = 300 mm, *L*′ = 400 mm, *h* = 100 mm and *b* = 100 mm). (**a**) Profile and (**b**) specimen. *F* is the applied vertical loading, and *L* is the distance between the two supports.

Concrete beams were employed in the three-point bending tests to elucidate the effect of strain rate on the tensile strength of concrete. The profile of the three-point bending test is presented in Figure 1, together with the casting concrete specimen. The applied force and arranged strain gauges are also shown in Figure 1. *F* is the applied vertical loading, and *L* is the distance between the two supports. *L*′, *b* and *h* is the length, width and height of the specimen. In this study, *L* = 300 mm, *L*′ = 400 mm, *h* = 100 mm and *b* = 100 mm is accepted.

### 2.2. Material Properties

The mixture proportion of the cement, sand and aggregate for the designed concrete beam was 430:482:1170 in the unit of kg/m^3^, where Ordinary Portland cement (P·O42.5) was used (See Table 1). A water-to-cement ratio of 0.3 was designed. One graded aggregate with a representative diameter of 25 mm and natural sand with an average size of 5 mm (fineness module was 3.2, coarse sand) were used. The size of the concrete sample was 150 × 150 × 150 mm^3^. The mold was removed after 24 h. After curing 28 days with constant room temperature and a relative humidity of 95%, the material properties, including compressive strength, and Young’s modulus of three cubic specimens 28 days after casting are given in Table 2.

In addition, the tensile strength of the cement mortar was measured with the three-point bending experiment, and the tensile strength of the cement mortar reached 2.16 MPa. The tensile strength of the aggregate was 15.38 MPa, provided by the manufacturer.

### 2.3. Loading and Measures

**Figure 2:**
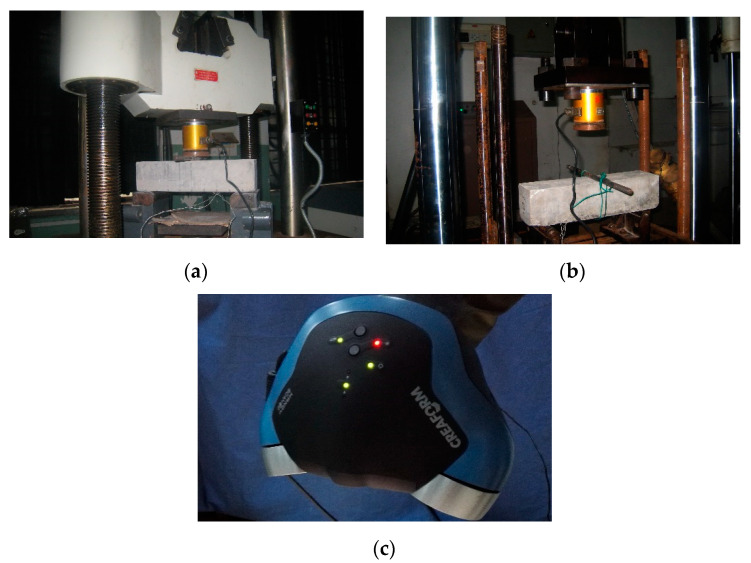
Experimental setup. (**a**) CSS200 Universal Material Testing Machine, (**b**) FDS300 Fatigue Testing Machine and (**c**) 3D laser scanner, Handy SCAN.

Two different loading types were considered, static loading and dynamic loading conditions, and the experiments were controlled by the nodal displacement where the vertical load was applied. The former experiments were carried out with a loading velocity of 0.1 mm/min on a CSS200 Universal Material Testing Machine Controlled by a computer with a hydraulic jack (see Figure 2a). The latter ones were conducted on an FDS300 Fatigue Testing Machine (Figure 2b) with three different loading velocities, 90 mm/min, 120 mm/min and 150 mm/min. A Dynamic Resistance Strain Apparatus DH-5920 was used in this study to record the applied force and strain during the loading. Each specimen was applied with two strain gauges (see Figure 1). Moreover, a Handy SCAN machine [25] (Figure 2c) was used to detect the areas of each concrete component on the fracture surface after the experiments. This instrument rebuilt a complex three-dimensional model and generated the line surface-volume data of the measured object by recording the information of 3D coordinates, reflectance and texture based on the principle of a laser rangefinder. The accuracy of Handy SCAN was up to 0.03 mm.

## 3. Experimental Results under Static Loading and Dynamic Loading

### 3.1. Static Loading Condition

Three concrete specimens were considered for the static loading conditions. After the specimen was assembled in the CSS200 universal material testing machine, the loading end approached the specimen at a low speed. When the reaction force of the loading point reached 0.03 kN, the universal material testing machine was loaded with a velocity of 0.1 mm/min. At that time, the DH-5920 started recording the vertical force and strain data until the specimen was destroyed.

**Figure 3:**
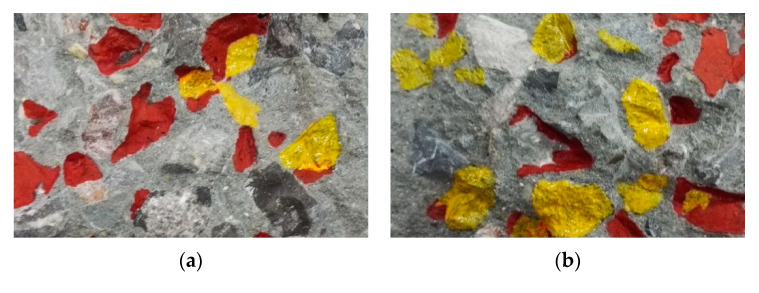

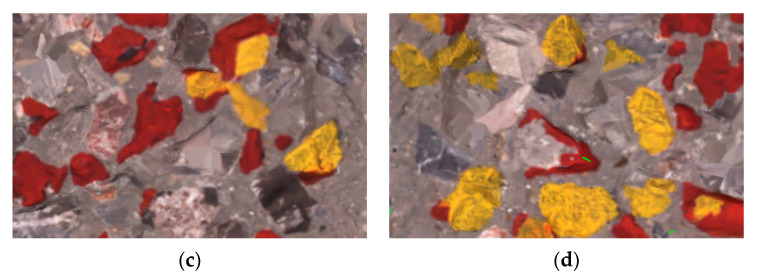
Real section profile (**a**,**b**) and 3D section profile generated by Handy SCAN (**c**,**d**) of the concrete specimen. (**a**) Side A, (**b**) Side B, (**c**) Side A and (**d**) Side B.

When the crack went through the concrete specimen during the experiment, two surfaces along the crack were obtained, called Side A and Side B. The crack surface was scanned by Handy SCAN to calculate the areas of different concrete components. Figure 3 elaborated the real section pictures (Figure 3a,b) and the 3D section profile obtained by Handy SCAN (Figure 3c,d) of one fractured beam. Different colors were marked on the cracked surface in order to distinguish the different components clearly, with yellow representing the aggregate and red the interface.

After comparison, the real cracked surface and 3D section profile of the cracked surface were basically the same. The obtained component area and the corresponding percentage under static loading were detailed in Table 3.

**Figure 4:**
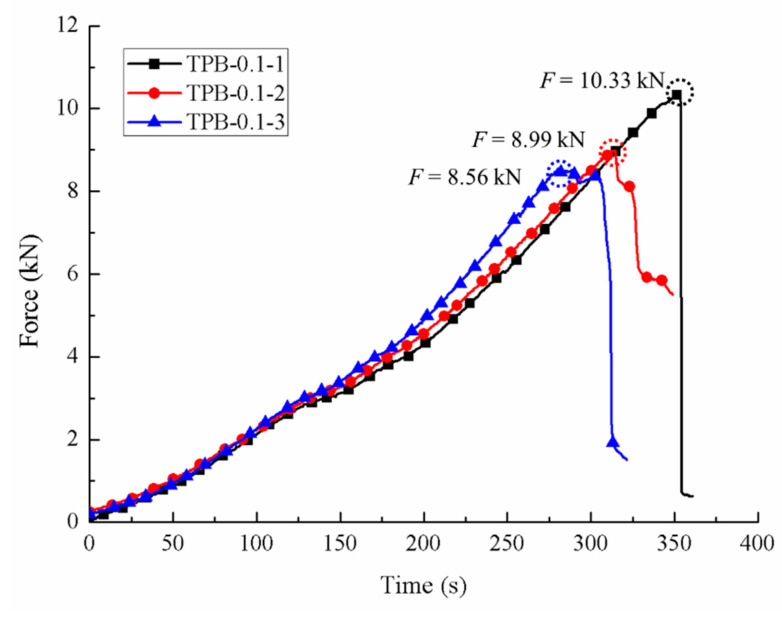
Force-time curves under the static loading conditions.

The force-time curves of three specimens were depicted in Figure 4 with the corresponding ultimate loads of 10.33 kN, 8.99 kN and 8.56 kN. The average value, 9.29 kN, was considered as the ultimate load of concrete for the static three-point condition. Considering the size of the beam, the Timoshenko beam theory was employed in the study. Hence, the static bending strength of the specimens can be easily derived as 4.65 MPa, 4.05 MPa and 3.85 MPa, respectively.

For concrete-like materials, it was accepted in this study [26] that the enhancement behavior in strength was due to the inhomogeneity of materials and the inertia force, which can be expressed as

(1) $${\dot{\varepsilon }}_{s}=1\times 10^{-6}s^{-1},$$

where $f_{m}$, $f_{a}$ and $f_{n}$ are the strengths of mortar, aggregate and interface, respectively, with the corresponding area of each component, $S_{m}$, $S_{a}$ and $S_{n}$. S is the total area of the crack surface. $f_{i}$ is the term concerning the effect of dynamic loading inertia, which is regarded as zero in the static condition. Based on the experiment, the strengths of the mortar and aggregate can be obtained as $f_{m}=2.16\;\mathrm{MPa}$ and $f_{a}=15.38\;\mathrm{MPa}$, separately.

Hence, the strength of the interface was derived as

(2) $$f_{n}=\frac{fS-f_{m}S_{m}-f_{a}S_{a}}{S_{n}}=1.73\;\mathrm{MPa}.$$

**Figure 5:**
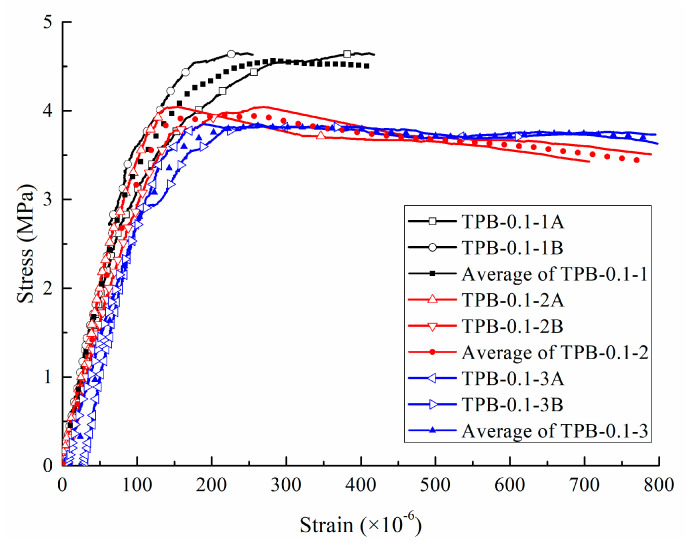
Stress-strain curves of three specimens under the static loading.

Figure 5 presents the stress-strain curves of three specimens for the static conditions considering two strain gauges on each specimen. It can be seen that, at the beginning of loading (within the range of 60 percent of the ultimate load), the stress-strain relation is roughly linear, representing the linear stage of the concrete fracture. Afterwards, the plasticity began in the specimen, and the gradient of the curve decreased before it reached the peak value, which was interpreted as the yield stress (*y*-axis) and yield strain (*x*-axis). Later, the stress decreased with the increasing strain, and the curve remained stably flat.

### 3.2. Dynamic Loading Condition

**Figure 6:**
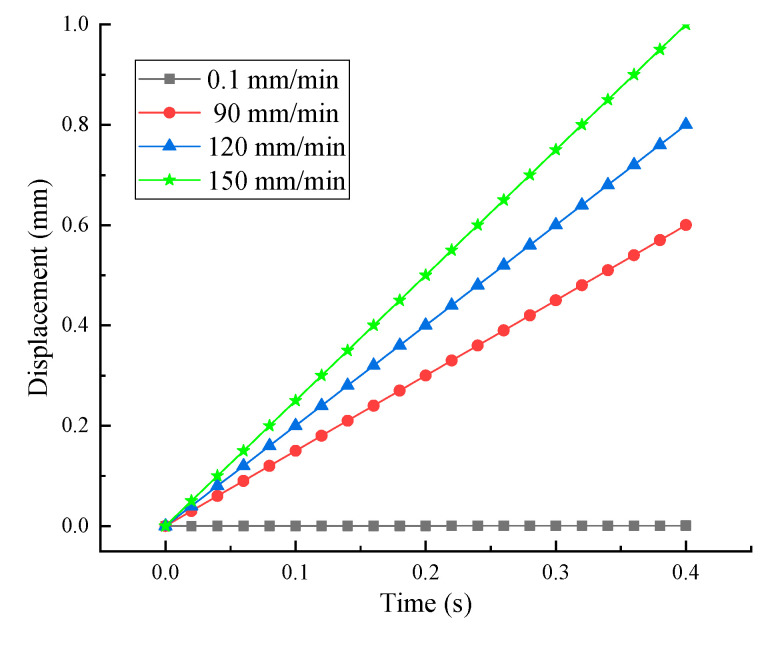
Loading rate profiles of the three-point bending experiments.

In order to investigate the effect of the loading rate on the tensile behavior of concrete, a series of three-point bending tests of concrete specimens under different loading velocities (0.1 mm/min, 90 mm/min, 120 mm/min and 150 mm/min) were conducted. The profile of the loading rate is detailed in Figure 6. All tests were divided into four groups, with three specimens for each group. The area percentages of different components under the static and dynamic loading conditions were obtained after the experiments, and the comparison results are listed in the following section.

The dynamic tests were conducted on an FDS300 fatigue testing machine with three different loading velocities, 90 mm/min, 120 mm/min and 150 mm/min. Similar to the static loading, the tests were controlled by the displacement of the loading point and the fatigue testing machine loaded with the designed velocity when the reaction force of the loading point reached 0.03 kN. The loading waveform was a triangular wave with an amplitude of 1 mm. Three frequencies were selected in the study, 0.37 Hz, 0.50 Hz and 0.63 Hz, resulting in the loading velocities of 90 mm/min, 120 mm/min and 150 mm/min, respectively. Three specimens were considered in each experimental condition.

**Figure 7:**
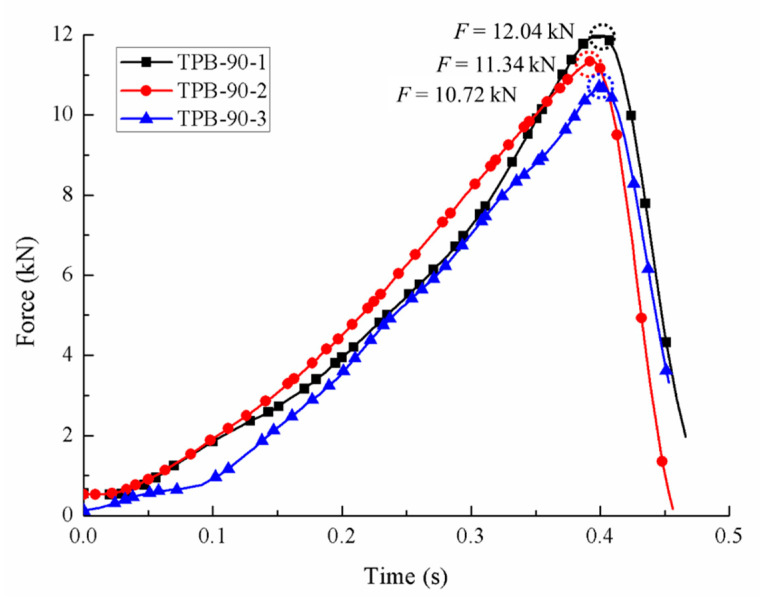
Force -time curves with the loading velocity of 90 mm/min.

**Figure 8:**
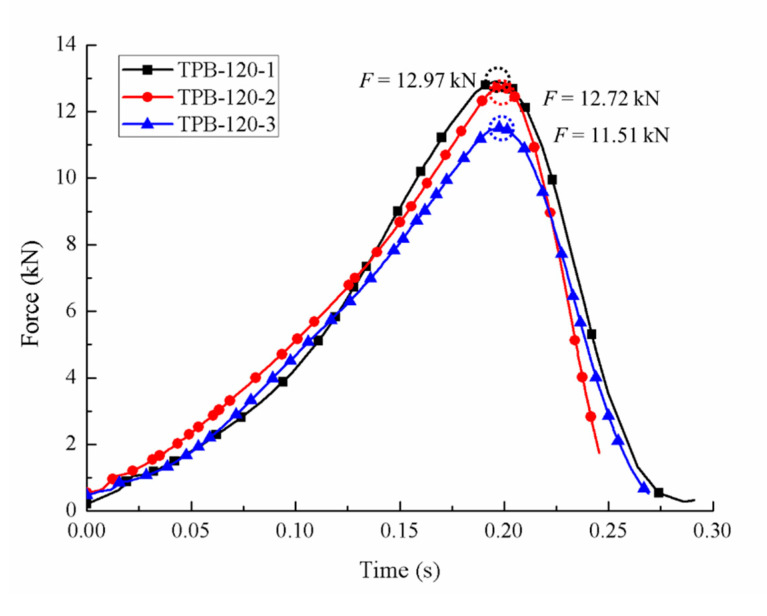
Force-time curves with the loading velocity of 120 mm/min.

**Figure 9:**
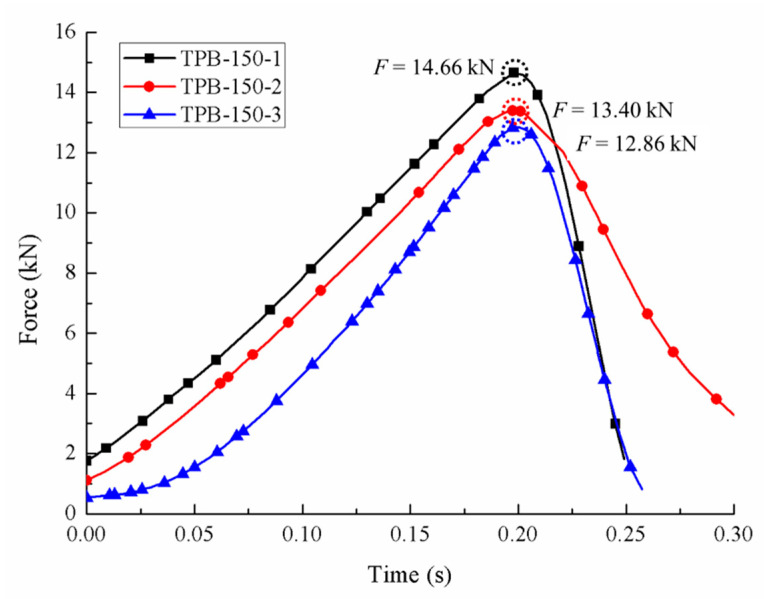
Force-time curves with the loading velocity of 150 mm/min.

**Figure 10:**
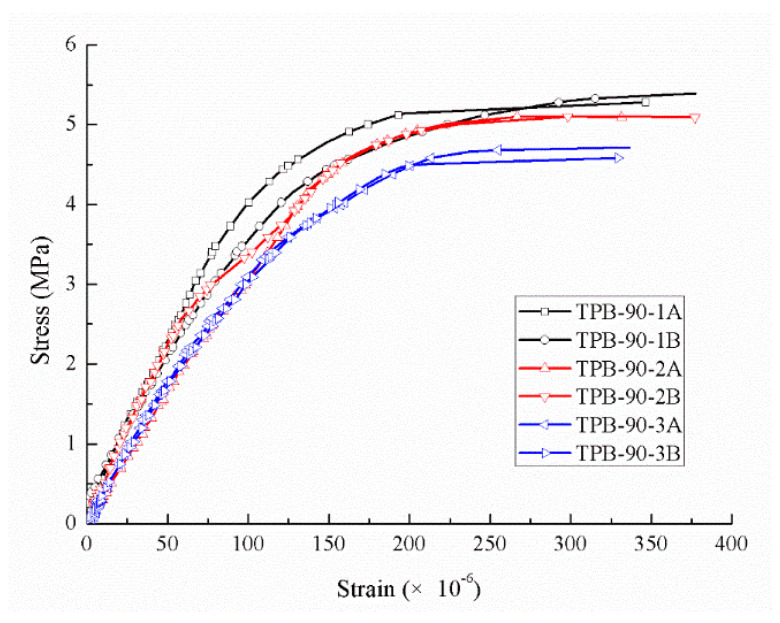
Stress-strain curves on the dynamic conditions (90 mm/min).

Compared to the static condition, the crack took less time from initiation to coalescence in the fast loading experiments, and the aggregates in the specimen were damaged more or less. The area proportions under different strain rates generated by the Handy SCAN are listed in Table 4. The force-time relations of three different dynamic loading velocities are depicted in Figure 7, Figure 8 and Figure 9, respectively, and the stress-strain curves under the loading velocity of 90 min/min are shown in Figure 10.

Figure 10 clearly elucidated that the relation between stress and strain was almost linear at the beginning of loading, almost the same as the static condition. Moreover, the gradient of the stress–strain curve increased with the increasing applied loading velocity, and it took less time for a beam to crack under faster loading.

The ultimate loads of the dynamic experiments are listed in Table 3. It is clear from the data that the ultimate loads under the dynamic condition were apparently higher than those under the static condition, and the concrete strength increased with the dynamic loading velocity.

## 4. Results and Discussion

To investigate the dynamic strength enhancement of the concrete due to the loading rate, the dynamic increase factors were observed through the experiment. The relation between the DIF and strain was established based on the experience and the experimental results. Furthermore, the effects of the aggregate and the inertia effect on the tensile strength of the concrete were studied in this section.

### 4.1. Dynamic Increase Factor

**Figure 11:**
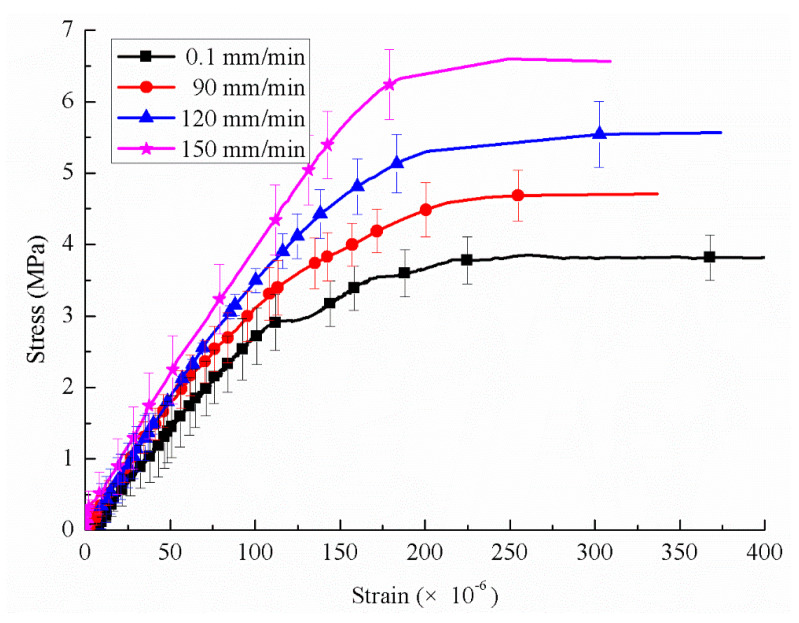
Stress-strain response of concrete under different loading velocities (skip every 5 points).

Figure 11 shows the stress-strain response of concrete under loading velocities that corresponded to different strain rates. It is obvious in the figure that the initial elastic modulus increases with the increasing strain rate. The tensile strength and stiffness appear distinctly sensitive to the strain rate.

**Figure 12:**
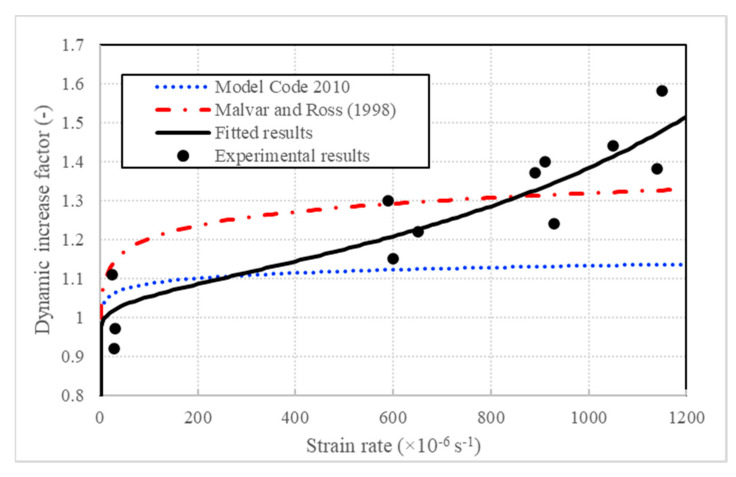
DIF (dynamic increase factor) curves and experimental data.

Based on the ultimate loads obtained from the experiments under different loading velocities and strain rates, the dynamic increase factors (DIFs) in tension are listed in Table 5. Hence, a relation between the DIF and strain rate was proposed as follows to predict the increase in strength with the increasing strain rate:

(3) $$T_{\mathrm{DIF}}=\frac{f_{t}^{dyn}}{f_{t}}=\kappa^{{\left[A\left(\kappa +B\right)\right]}^{C}},$$

where *A*, *B* and *C* are rate sensitive coefficients, and $\kappa =lg\left(\dot{\varepsilon }/{\dot{\varepsilon }}_{stat}\right)$ and ${\dot{\varepsilon }}_{stat}=10^{-6}s^{-1}$. According to the static and dynamic experimental data, the static ultimate tensile load of concrete was $f_{t}=9.29\;\mathrm{kN}$, and the fitted coefficients were A=−6.06, B=−3.46 and C=−1.19. Moreover, the proposed relation of Equation (3) was compared with the Model Code 2010 recommended strain-rate-induced strength increase equation [27] and the modified CEB (Comité Euro-International du Béton) for the strain-rate effect on concrete strength [16]. The curves, together with the experimental data, are elaborated in Figure 12, and the comparison of the fitted DIF and the experimental results are detailed in Table 6.

It can be seen from Figure 12 and Table 6 that, in the region between ${\dot{\varepsilon }}_{stat}$ and 10^−3^ s^−1^, the proposed formula is slightly different from the modified CEB equation and the relation in Model Code for Concrete Structures 2010. The relation in the Model Code for Concrete Structures 2010 underestimated the dynamic strength enhancement ability of concrete in the region between ${\dot{\varepsilon }}_{stat}$ and 10^−3^ s^−1^, while the equation proposed by Malvar and Ross overvalued the strain rate sensitive effect partly. Hence, this formula is proper to predict the tensile strength enhancement effect within the strain rate.

### 4.2. Effect of Aggregate on Dynamic Strength

As observed in the experiments under different strain rates, the specimen fractured more thoroughly under the rapid loading rate, and the aggregates in the specimen were damaged to some extent. The crack even passed through the center of the aggregates at a high strain rate. As calculated in Section 4.1, the strength of the aggregate was much greater than that of the mortar and interface; hence, the effect of the aggregate on the dynamic strength was studied in this section.

The contribution of the aggregate to the concrete strength is presented in Table 5. The increasing area percentage of the aggregate with the increasing strain rate on the crack surface demonstrated the aforementioned fact that the higher the strain rate, the greater the destruction of aggregate.

Define the ratio of the contribution of the aggregate to the dynamic strength of the concrete, $f_{a}^{dyn}S_{a}^{dyn}/S$, to the contribution of aggregate in the static tests, $\frac{f_{a}S_{a}}{S}=2.52\;\mathrm{MPa}$, as the strengthen coefficient, $D_{a}$. The relationship of the coefficient and the strain rate was proposed as follows:

(4) $$D_{a}=\exp\left\{{\left[a\times \left(lg\left(\dot{\varepsilon }\right)+b\right)\right]}^{c}\right\}\;10^{-6}\leq \dot{\varepsilon }\leq 10^{-3},$$

where *a*, *b* and *c* are fitting parameters for the strengthen coefficient. a=−4.04, b=2.52 and c=−2.24.

**Figure 13:**
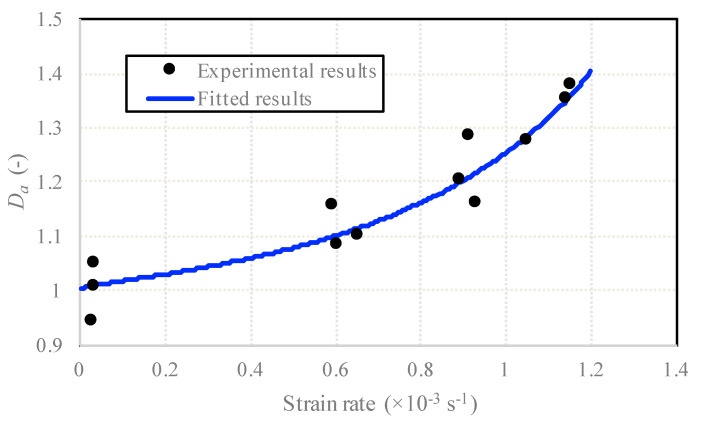
The strengthen coefficient-strain rate with respect to the strain rate.

Figure 13 depicts the strengthen coefficient–strain rate curve and the data obtained from the experiments. The assumed relationship showed good consistency with the experimental results.

### 4.3. Effect of Inertia on Dynamic Strength

**Figure 14:**
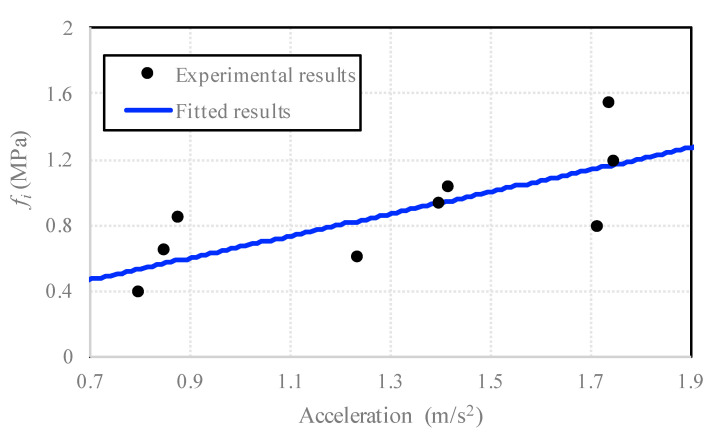
The dynamic inertia contribution with respect to the acceleration.

According to Equation (1), the terms produced by the dynamic loading inertia, $f_{i}$, under different strain rates were calculated and presented in Table 5. Compared to the contribution of the aggregate (see Table 4), the dynamic inertia term was small within the range of the strain rate from 1 × 10^−6^ s^−1^ to 1 × 10^−3^ s^−1^. However, the inertia term continued increasing and became more important with the increase of the strain rate. Finally, the experimental and fitted results of the dynamic inertia contribution with respect to the acceleration are shown in Figure 14, and an empirical formula of loading acceleration and the inertia contribution to the dynamic strength was observed as

(5) $$f_{i}=\beta \ddot{u,}\;\beta =0.67.$$

## 5. Numerical Simulation on the Mesoscopic Scale

**Figure 15:**
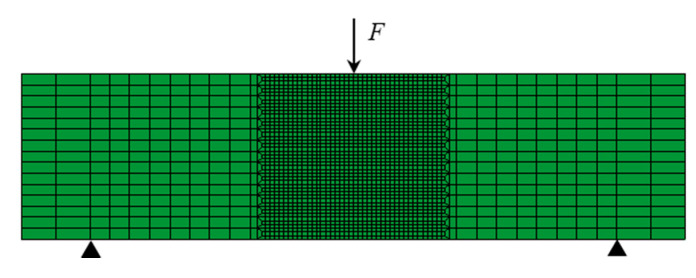
Three-point bending model for the finite element simulation.

**Figure 16:**
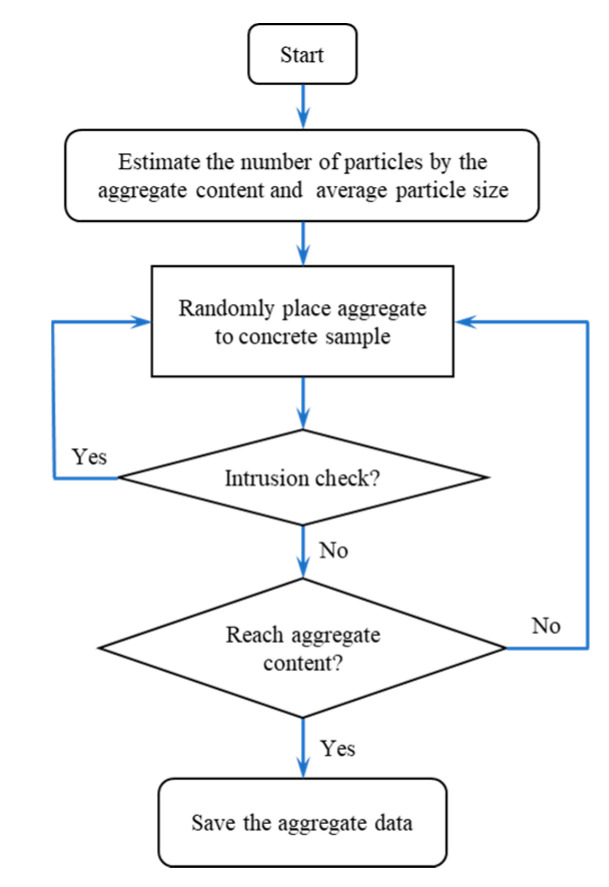
The flowchart of the geometric modeling.

**Figure 17:**
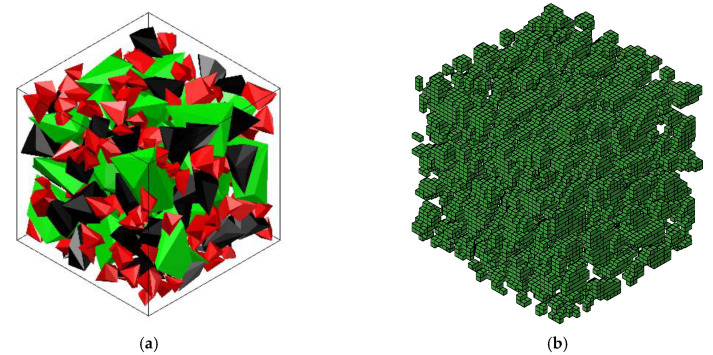
Schematic diagram of the aggregate placement and the aggregate element for simulation. (**a**) Aggregate geometry and (**b**) aggregate elements.

According to the mechanical characteristics of the three-point bending beam, the zone of 100 mm in the middle of the beam is considered as the pure bending section where endures the largest bending moment and the failure usually occurs. Hence, in the numerical simulation, the central part (100 mm × 100 mm × 100 mm) was selected as the heterogeneous section (see Figure 15). Based on the section profile obtained by the Handy SCAN and the concrete mix proportion, the 3D-RAS proposed by Sun [28] was employed in this numerical study to generate the polyhedral model for the numerical simulation on the mesoscopic scale. The geometric modeling procedure can be seen in Figure 16. It is very difficult to divide the finite element mesh strictly according to the aggregate, interface and mortar of the polyhedral model in the 3D numerical specimen. The background grid method [29] was adopted to distinguish each phase in the concrete and build the numerical model. The schematic diagram of aggregate geometry and the aggregate element for simulation are elaborated in Figure 17. Hence, the number of the aggregate elements in the finite element mesh was 21,548, the interface was 18,784 and the mortar was 23,668. Moreover, the material parameters of the aggregate, interface and mortar used in the numerical analysis are listed in Table 7.

The finite element simulation was conducted with the Abaqus software (manufactured by Dassault SIMULIA, powered by the 3DEXPERIENCE^®^ platform, USA). The plastic damage model proposed by Lubliner et al. [30] and Lee et al. [31], which was suitable for cyclic loading simulations, was adopted, and the softening effect of concrete-like materials was considered in this study. During the simulation, the loading was applied on the center of the 3D model, and the loading velocity increased to the amplitude in 10^−4^ s. For brevity, only the loading velocities of 0.1 mm/min and 150 mm/min were considered. The rate effect of concrete obtained by the experiments (Equation (3)) was embedded in the Abaqus simulations.

**Figure 18:**
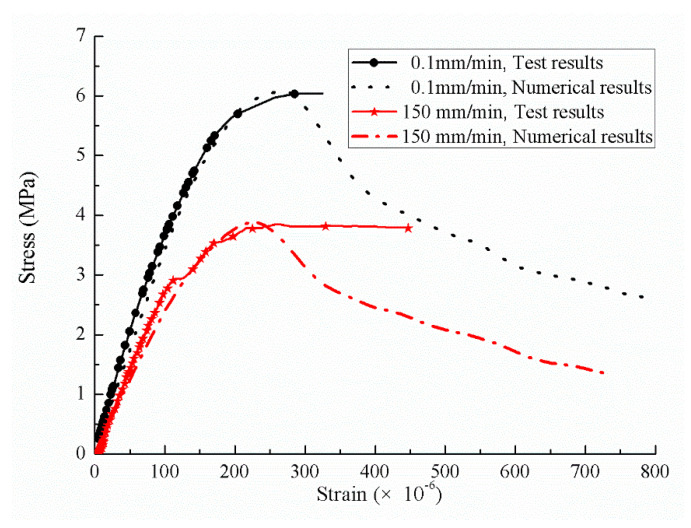
Stress-strain curves comparison under different loading rates.

The stress-strain curves calculated by the finite element analysis and the experiments are illustrated in Figure 18. As clearly observed in Figure 18, the numerical results were almost the same as those of the experimental results, especially the upward section of the stress-strain curves and the critical strain with the loading velocities of 0.1 mm/min and 150 mm/min.

## 6. Conclusions

In this study, the effect of strain rate on the concrete was investigated with the three-point bending tests. Four sets of three-point bending tests of concrete beams were carried out under different strain rates to examine the rate-sensitive behavior of concrete-like materials. After the experiments, a novel 3D laser scanner, Handy SCAN, was employed to detect the areas of interface, mortar and aggregate on the fracture surface. Based on the experimental data, a dynamic concrete strength model was proposed. Besides, an empirical relationship between the strain rate and DIF was established under the strain rate range of 10^−6^ s^−1^ and 1.5 × 10^−3^ s^−1^.The following conclusions can be drawn:

(1) The inherent inhomogeneity of the materials and the inertial effects were considered as the main factors responsible for the strength enhancement of the concrete. According to the experimental results of the stress-strain curves, the tensile strength and stiffness appeared distinctly sensitive to the strain rate.
(2) A relation between DIF in the tension and strain rate was proposed to predict the increase in strength with the increasing strain rate, especially under the range of 10^−6^ s^−1^ and 1.5 × 10^−3^ s^−1^.
(3) The aggregate played an important role in the dynamic strength enhancement; the higher the strain rate, the greater the destruction of the aggregates. The inertia effect was quantified and increased linearly with the acceleration.
(4) The obtained experimental results were employed in the finite element analysis of the concrete beams, and the numerical results consisted well with the experimental results.

## Figures and Tables

**Table 1:** Mixture proportion of concrete in the unit of kg/m^3^.

Water	Cement	Sand	Aggregate (Representative Particle Size: 25 mm)
129	430	482	1170

**Table 2:** Material properties of the concrete.

Nominal Strength (MPa)	Compressive Strength (MPa)		Tensile Strength (MPa)		Young’s Modulus (MPa)	
	Sample Value	Representative Value	Sample Value	Representative Value	Sample Value	Representative value
30	30.6	30.2	3.56	3.54	36,400	35,900
	28.9		3.48		35,200	
	31.1		3.59		36,100	

**Table 3:** Component area and the corresponding percentages under static loading.

Specimen	Strain Rate (× 10^−6^ s^−1^)	Area (mm^2^)			Percentage of Area (%)		
		Aggregate	Interface	Mortar	Aggregate	Interface	Mortar
TPB-0.1-1	25	1811.06	3992.07	5930.86	15.43	34.02	50.54
TPB-0.1-2	28	1966.26	3712.09	6261.85	16.46	31.10	52.44
TPB-0.1-3	31	2085.44	3984.22	6053.09	17.20	32.87	49.93

**Table 4:** Area percentage and ultimate loads under different strain rates.

Specimen	Strain Rate (× 10^−6^ s^−1^)	Area (mm^2^)			Percentage of Area (%)			Ultimate Load (kN)
		Aggregate	Interface	Mortar	Aggregate	Interface	Mortar	
TPB-90-1	590	2346.18	3816.82	6221.44	18.94	30.82	50.24	12.04
TPB-90-2	650	2140.36	3844.54	5898.61	18.01	32.35	49.64	11.34
TPB-90-3	600	2158.75	3592.93	6421.50	17.73	29.52	52.75	10.72
TPB-120-1	910	2589.93	2952.06	6776.41	21.02	23.96	55.01	12.97
TPB-120-2	890	2392.52	3602.42	6134.63	19.72	29.70	50.58	12.72
TPB-120-3	930	2239.58	3998.46	5163.36	18.98	34.73	46.29	11.51
TPB-150-1	1140	2696.96	4221.81	5259.12	22.15	34.67	43.19	14.66
TPB-150-2	1050	2355.97	2871.46	6068.22	20.86	25.42	53.72	13.40
TPB-150-3	1150	2502.51	3662.71	4921.66	22.57	33.04	44.39	12.86

**Table 5:** Contribution of aggregate and dynamic inertia to the concrete strength under dynamic conditions. (DIF: dynamic increase factor, *f* is the dynamic strength, $\ddot{u}$ is the acceleration and *f_i_* is the strength contributed by the inerita).

Load Velocity (mm/min)	Experimental DIF	Strain Rate (× 10^−6^ s^−1^)	f (MPa)	$f_{a}S_{a}/S$ (MPa)	$\ddot{u}$ (m/s^2^)	$f_{i}$ (MPa)
90	1.30	590	5.42	2.91	0.88	0.83
	1.22	650	5.10	2.77	0.85	0.64
	1.15	600	4.82	2.73	0.80	0.39
120	1.40	910	5.80	3.23	1.40	0.92
	1.37	890	5.72	3.03	1.42	1.03
	1.24	930	5.18	2.92	1.24	0.60
150	1.38	1140	6.60	3.41	1.72	0.79
	1.44	1050	6.03	3.21	1.75	1.18
	1.58	1150	5.79	3.47	1.74	1.54

**Table 6:** Comparison of the obtained DIF and the fitted results.

Specimen	T1	T2	T3	T4	T5	T6	T7	T8	T9	T10	T11	T12
Test	0.92	0.97	1.11	1.15	1.22	1.24	1.30	1.37	1.38	1.40	1.44	1.58
Model code 2010	1.06	1.06	1.05	1.29	1.12	1.13	1.12	1.13	1.14	1.13	1.13	1.13
Malvar	1.14	1.14	1.13	1.26	1.29	1.31	1.29	1.31	1.33	1.31	1.32	1.33
This paper	1.01	1.02	1.01	1.20	1.22	1.34	1.20	1.33	1.47	1.33	1.41	1.47

**Table 7:** Material parameters used in the analysis.

Material	Young’s Modulus (MPa)	Poisson’s Ratio	Fracture Energy (N/m)	Tensile Strength (MPa)
Aggregate	80	0.16	140.4	15.38
Mortar	30	0.22	71.3	2.16
Interface	22	0.16	46.8	1.73
Concrete	30	0.17	-	-

## References

[1] Bischoff P.H., Perry S.H. (1995). Impact behavior of plain concrete loaded in uniaxial compression. J. Eng. Mech..

[2] Zhang Y., Xu Y., Zheng Y., Fernandez-Rodriguez E., Sun A., Yang C., Wang J. (2019). Multi objective Optimization Design and Experimental Investigation on the Axial Flow Pump with Orthogonal Test Approach. Complexity.

[3] Chen X., Wu S., Zhou J. (2013). Experimental and modeling study of dynamic mechanical properties of cement paste, mortar and concrete. Constr. Build. Mater..

[4] Bischoff P.H., Perry S.H. (1991). Compressive behavior of concrete at high strain rates. Mater. Struct..

[5] Mazars J., Millard A. (2010). Dynamic Behavior of Concrete and Seismic Engineering.

[6] Abrams D.A. (1917). Effect of rate of application of load on the compressive strength of concrete. ASTM J..

[7] Ross C.A., Tedesco J.W., Kuennen S.T. (1995). Effects of strain rate on concrete strength. Mater. J..

[8] Guo Y., Gao G., Lin J., Shim V.P.W. (2019). Quasi-static and Dynamic Splitting of High-strength Concretes-Tensile Stress-strain Response and Effects of Strain Rate. Int. J. Impact Eng..

[9] Li Q., Meng H. (2003). About the dynamic strength enhancement of concrete-like materials in a split Hopkinson pressure bar test. Int. J. Solids Struct..

[10] Chen J.J., Guo B., Liu H., Liu H.B. (2014). Dynamic Brazilian Test of Brittle Materials Using the Split Hopkinson Pressure Bar and Digital Image Correlation. Strain.

[11] Zhou X., Hao H. (2008). Modelling of compressive behaviour of concrete-like materials at high strain rate. Int. J. Solids Struct..

[12] Lv T., Chen X., Chen G. (2017). Analysis on the waveform features of the split Hopkinson pressure bar tests of plain concrete specimen. Int. J. Impact Eng..

[13] Zhao H., Gary G. (1997). A new method for the separation of waves. Application to the SHPB technique for an unlimited duration of measurement. J. Mech. Phys. Solids.

[14] Reinhardt H.W. Testing and monitoring techniques for impact and impulse loading of concrete structures. Proceedings of the RILEM Symposium on Impact and Impulsive Loading of Concrete Structures.

[15] Comité Euro-International du Béton (1993). CEB-FIP Model Code 1990.

[16] Malvar L.J., Ross C.A. (1998). Review of strain rate effects for concrete in tension. Mater. J..

[17] Grote D.L., Park S.W., Zhou M. (2001). Dynamic behavior of concrete at high strain rates and pressures: I. experimental characterization. Int. J. Impact Eng..

[18] Brace W.F., Jones A.H. (1971). Comparison of uniaxial deformation in shock and static loading of three rocks. J. Geophys. Res..

[19] Qi C.Z., Qian Q.H. (2003). Physical mechanism of dependence of material strength on strain rate for rock-like material. Chin. J. Rock Mech. Eng..

[20] Lu Y., Li Q. (2011). About the dynamic uniaxial tensile strength of concrete-like materials. Int. J. Impact Eng..

[21] Hentz S., Donze F.V., Daudeville L. (2004). Discrete element modelling of concrete submitted to dynamic loading at high strain rates. Comput. Struct..

[22] Kim D.J., Sirijaroonchai K., El-Tawil S., Naaman A.E. (2010). Numerical simulation of the split hopkinson pressure bar test technique for concrete under compression. Int. J. Impact Eng..

[23] Weerheijm J. (1992). Concrete under Impact Tensile Loading and Lateral Compression. Ph.D. Thesis.

[24] Dang F., Pan F., Jiao K., Shi J. (2015). Mechanism for enhancement of dynamic strength and failure model of nonuniform brittle materials. Earthq. Eng. Eng. Dyn..

[25] Li W., Mitchell L. (1995). Laser scanning system testing-Errors and improvements. Measurement.

[26] Posiadala B. (1997). Free vibrations of uniform Timoshenko beams with attachments. J. Sound Vib..

[27] Betonbau F. (2013). Fib Model Code for Concrete Structures 2010.

[28] Du C., Sun L. (2006). Numerical simulation of concrete aggregates with arbitrary shapes and its application. J. Hydraul. Eng..

[29] Hao O., Chen X. (2020). 3D meso scale modeling of concrete with a local background grid method. Constr. Build. Mater..

[30] Lubliner J., Oliver J., Oller S., Oñate E. (1989). A plastic-damage model for concrete. Int. J. Solids Struct..

[31] Lee J., Fenves G. (1998). Plastic-Damage Model for Cyclic Loading of Concrete Structures. J. Eng. Mech..

